# IL-6-mediated migration of human metastatic melanoma cells is reduced by simvastatin treatment

**DOI:** 10.1186/2050-6511-13-S1-A66

**Published:** 2012-09-17

**Authors:** Christine Wasinger, Christoph Minichsdorfer, Martin Hohenegger

**Affiliations:** 1 Institute of Pharmacology, Center for Physiology and Pharmacology, Medical University of Vienna, 1090 Vienna, Austria; 2 Department of Oncology, Medical University of Vienna, 1090 Vienna, Austria

## Background

In 1987 the HMG-CoA reductase inhibitors, statins, were first marketed and are now widely used as well-tolerated therapeutics for hypercholesterolemia. High interleukin 6 (IL-6) plasma levels in melanoma patients are linked to a higher tumour burden and reduced overall survival. We have recently shown that simvastatin triggers apoptosis in human metastatic melanoma cells which is associated with concentration-dependent changes in autocrine IL-6 secretion. Here, we investigated IL-6 signalling with respect to proliferation and migration in human metastatic melanoma cells under statin application.

## Methods

For this approach, human metastatic melanoma cells (A375, 518a2) were used for quantification of surface expression of the IL-6 receptor (IL-6-R/gp130) and for cell cycle with FACS analysis. Additionally, migration assays were carried out with simvastatin and/or IL-6 administration over time.

## Results

Increasing concentrations of simvastatin led to morphological changes and detachment of the melanoma cells. After reseeding of the detached cells, A375 cells had a normal cell cycle profile while 518a2 cells underwent apoptosis resulting in cell death. Moreover, simvastatin treatment enhanced the surface expression of the IL-6-R and the gp130 subunit in a time- and concentration-dependent manner. Both cell lines responded to IL-6 treatment with increased proliferation and migration which was inhibited by simvastatin.

## Conclusion

We demonstrate that simvastatin up-regulates the IL-6 pathway on the level of the heteromeric IL-6 receptor. Although increased IL-6 receptor expression would imply a stronger IL-6 signal, this is not seen in the presence of simvastatin. A novel therapeutic concept for simvastatin may emerge from the suppression of the IL-6-mediated proliferation and migration in metastatic melanoma cells.

